# Intake of high fructose corn syrup sweetened soft drinks is associated with prevalent chronic bronchitis in U.S. Adults, ages 20–55 y

**DOI:** 10.1186/s12937-015-0097-x

**Published:** 2015-10-16

**Authors:** Luanne Robalo DeChristopher, Jaime Uribarri, Katherine L. Tucker

**Affiliations:** 1 Biochemistry, Molecular Biology, NY Medical College, Valhalla, NY USA; 2 Department of Medicine, the Icahn School of Medicine, New York, NY USA; 3 Department of Clinical Laboratory and Nutritional Sciences, University of Massachusetts, Lowell, MA USA; 4 P.O. Box 5542, Eugene, OR 97405 USA

**Keywords:** Chronic bronchitis, COPD, Advanced glycation end-products, Fructose, Fructose malabsorption, Fructositis, HFCS, Excess free fructose, EnFruAGE, EFF, High fructose corn syrup

## Abstract

**Background:**

High fructose corn syrup (HFCS) sweetened soft drink intake has been linked with asthma in US high-schoolers. Intake of beverages with excess free fructose (EFF), including apple juice, and HFCS sweetened fruit drinks and soft drinks, has been associated with asthma in children. One hypothesis for this association is that underlying fructose malabsorption and fructose reactivity in the GI may contribute to in situ formation of enFruAGEs. EnFruAGEs may be an overlooked source of advanced glycation end-products (AGE) that contribute to lung disease. AGE/ RAGEs are elevated in COPD lungs. EFF intake has increased in recent decades, and intakes may exceed dosages associated with adult fructose malabsorption in subsets of the population. Intestinal dysfunction has been shown to be elevated in COPD patients. The objective of this study was to investigate the association between HFCS sweetened soft drink intake and chronic bronchitis (CB), a common manifestation of COPD, in adults.

**Methods:**

Design: In this cross sectional analysis, the outcome variable was self-reported existing chronic bronchitis or history of CB. Exposure variable was non-diet soda. Rao Scott Ҳ^2^ was used for prevalence differences and logistic regression for associations, adjusted for age, sex, race-ethnicity, BMI, smoking, exposure to in-home smoking, pre-diabetes, diabetes, SES, total energy and total fruits and beverages consumption.

**Setting:**

Data are from the National Health and Nutrition Examination Survey 2003–2006.

**Subjects:**

2801 adults aged 20–55 y.

**Results:**

There was a statistically significant correlation between intake of non-diet soft drinks and greater prevalence and odds of chronic bronchitis (*p* < 0.05). Independent of all covariates, intake of non-diet soda ≥5 times a week (vs. non/low non-diet soda) was associated with nearly twice the likelihood of having chronic bronchitis (OR = 1.80; *p* = 0.047; 95 % CI 1.01–3.20).

**Conclusions:**

HFCS sweetened soft drink intake is correlated with chronic bronchitis in US adults aged 20–55 y, after adjusting for covariates, including smoking. Results support the hypothesis that underlying fructose malabsorption and fructose reactivity in the GI may contribute to chronic bronchitis, perhaps through in situ formation of enFruAGEs, which may contribute to lung disease. Longitudinal and biochemical research is needed to confirm and clarify the mechanisms involved.

## Background

Chronic obstructive pulmonary disease (COPD) is the third leading cause of death in America [1]. COPD refers to two lung diseases that cause airflow blockage and breathing-related problems. It includes emphysema, and chronic bronchitis. While emphysema is primarily caused by smoking and is characterized by a permanent enlargement of the airways and destruction of the walls of the alveoli, [2] chronic bronchitis, is caused by overproduction and hyper-secretion of mucus by goblet cells, which leads to worsening airflow obstruction, epithelial remodeling, and alteration of airway surface tension, predisposing to collapse. [3] Because the mucus is thick and abundant, it is often difficult for a person with chronic bronchitis to expel it. Large amounts of thick mucus create an environment that is conducive to bacterial growth. Therefore, lung infections are common and frequent among people with chronic bronchitis [1–3].

In 2011, 6.5 % of adults (approximately 13.7 million) reported having been diagnosed with COPD [4]. However, earlier reports indicate that close to 24 million US adults have evidence of impaired lung function, suggesting an under diagnosis of COPD [5]. Prevalence data that distinguishes between smoking-related emphysema and mucus hypersecretion related chronic bronchitis is unavailable.

According to the American Lung Association (ALA), while asthma is a chronic childhood disease affecting 10% of children, COPD is a chronic adult disease. The ALA reports that children who suffered from severe, persistent asthma are nearly 32 times more likely to develop COPD in adulthood. Many patients with long-standing asthma develop airway remodeling that causes a chronic irreversible airflow obstruction, or COPD [6]. Recent research has suggested that emphysema and chronic bronchitis, traditionally considered to be entities that overlap within COPD, may be distinct disorders. Researchers found that while smoking is a leading risk factor in COPD, there exists considerable heterogeneity among different COPD phenotypes. For example, patients with self-reported chronic bronchitis were significantly younger and less likely to be a current or former smoker as compared with self-reported COPD patients [7].

Anecdotal evidence links high fructose corn syrup (HFCS) consumption with chronic bronchitis and asthma [8]. Recent research has linked HFCS sweetened soft drink intake with asthma in US high-schoolers [9]. High intakes of excess free fructose (EFF) beverages, including apple juice, and HFCS sweetened fruit drinks and soft drinks have been associated with asthma in children [10]. The childhood asthma study was motivated by the previously proposed “enFruAGEs fructositis hypothesis” [8] - that underlying fructose malabsorption and associated fructose reactivity in the gastrointestinal tract (GI) may contribute to in situ formation of enFruAGEs - an overlooked source of advanced glycation end-products (AGEs) - that are known to be associated with lung inflammation and pathophysiology [11].

Recent murine-based research provides evidence of AGEs formation in the jejunum [12, 13], and the receptor of AGEs (RAGE) has emerged as a mediator of asthma [14]. Increased staining for both AGEs and RAGE in lung tissues of COPD patients raised the possibility that AGEs-RAGE interaction may have a role in the pathogenesis of chronic bronchitis, as a subcomponent of COPD, which is unrelated to smoking [15].

There is also evidence that more excess free fructose is being consumed than has been assumed. Results of a 2014 study indicate popular soda beverages are sweetened with an HFCS variant that is 60 % fructose and 40 % glucose, rather than the 55/45 formula that is generally recognized as safe [16]. Different varieties of fruit drinks with high apple juice content were found to contain a 67/33 % combination of fructose and glucose monomers [16]. These findings raise the possibility that daily excess free fructose intakes exceed dosages that have been correlated with fructose malabsorption [17–22]. Notably, sucrose and equal amounts of fructose and glucose monomers are not associated with adult fructose malabsorption [23].

There is growing clinical interest in secondary organ manifestations of COPD, particularly in the GI tract [24]. There is recent evidence that a high percentage of COPD patients have GI disturbance including abdominal bloating, and flatulence [25]. COPD sufferers have nearly three times the risk of Crohn’s disease [26]. Biomarker analysis has provided evidence of increased permeability of the small intestine and colon of COPD patients [27]. Researchers proposed that intestinal compromise should be considered as a component of COPD and that COPD should be viewed as a multisystem disorder [27]. Notably, there is considerable overlap between GI symptoms in COPD and GI symptoms in fructose malabsorption including gas, bloating, increased flatulence, and abdominal pain [17–22].

The objective of this study was to test the hypothesis that increasing HFCS sweetened soft drink intake is associated with prevalent chronic bronchitis, as a subcomponent of COPD unrelated to smoking, based on the mechanistic “intestinal enFruAGEs fructositis” hypothesis -- that FM underlies the intestinal in situ formation of pro-inflammatory enFruAGEs associated with lung disease. In this cross sectional epidemiological study, we studied the association between chronic bronchitis prevalence and intake frequency of non-diet soft drinks - the high excess free fructose beverage most consumed by US adults [28, 29]. We used a large nationally representative survey dataset - the National Health and Nutrition Examination Survey (NHANES) - that included data obtained from a food frequency questionnaire for years 2003–2006. During this data collection period, HFCS was the predominant sweetener in US soft drinks [29].

## Methods

### Sample and survey administration

We used data from the 2003–2006 U.S. National Health and Nutrition Examination Survey (NHANES) [30]. NHANES are designed to assess the health and nutritional status of adults and children in the United States. These surveys are unique in that they combine interviews and physical examinations, as well as dietary intake. In 2003–06, a food intake frequency questionnaire (FFQ) was added to assess usual intake of specific foods and food groups. Responses to the FFQ rather than 24 h recall data were used in this analysis, because the objective of the study was to analyze long-term patterns of intake rather than detail on specific days. Strong and consistent relationships have been reported between reported FFQ frequency of food and food-group consumption and probability of consumption on 24-h recalls [31]. Food frequency questionnaires are frequently used as reliable sources of dietary patterns and food intake information in epidemiological research [9, 32–35].

NHANES uses a complex sampling design and constructs sample weights to produce nationally representative data. Therefore sample weights are provided to account for oversampling. The weights used in this study were those provided within the food frequency data files. In the 2003–06 survey periods, the sampling fractions and screener rates were set to over-sample certain groups (i.e. low income persons, adolescents, the elderly, non-Hispanic blacks, and Mexican Americans) to increase the ability to obtain more precise estimates for these groups [36]. Therefore, all statistics and summary tables are appropriately weighted to account for oversampling. The number of observations, within summary Table 1, reflects the weighted number of respondents for each variable.

**Table 1:** Characteristics of adults, aged 20–55 y in the NHANES 2003–2006

N	Number of subjects	2801
Age	(y, mean ± SD)	36.5 ± 8.6
Sex	(% male)	51.3
Race/ethnicity (%)		
	Non-Hispanic White	65.7
	Non-Hispanic Black	14.5
	Mexican American	10.8
	Other Hispanic	2.8
	Other	6.1
BMI	(mean ± SD)	27.9 ± 5.6
Energy intake (kcal, (mean ± SD)		2352 ± 744
Chronic Bronchitis (%)		
No		93.9 %
Yes		06.1 %

Information of chronic bronchitis status was available for adults, aged 20 y and older. For this analysis, the focus was on individuals aged 20 through 55 y, as existing research indicates that soft drink consumption is highest among young adults and begins to decline as adults approach their latter 50’s [37]. There were 2,801 adults aged 20–55 y with complete responses to questions regarding non-diet soft drink intake, demographic data and chronic bronchitis status.

### Variables

The outcome variable was self-reported current or prior chronic bronchitis. On the NHANES questionnaire, this was asked as “Has a doctor or other health professional ever told you that you have chronic bronchitis?” The exposure to non-diet soda was obtained from the FFQ questions: How often did you drink 1) soft drinks, soda, or pop in the summer? and 2) soft drinks, soda, or pop the rest of the year? Additional questions clarified how often soft drinks were diet or sugar-free, or caffeine free [30].

The average daily frequency of non-diet soft drinks over the past year was calculated by summing individual values for caffeinated and caffeine-free non-diet soft drinks-in-the-summer and rest-of-year. The NHANES utilized specialized software [Diet-Calc] to assign frequencies to responses from the FFQ using algorithms as follows: Never = 0; 1 time per mon or less = 0.03; 2–3 times per mon = 0.08; 1–2 times/ wk = 0.21; 3–4 times/ wk = 0.5; 5–6 times/ wk = 0.79; 1 time/ d = 1; 2–3 times/ d = 2.5 [30]. Intake data were combined to establish new intervals for analysis purposes as follows: ≤ 2–3 times/ mon as the reference group; 1–4 times/ wk; and ≥ 5 times/wk.

Adjustment variables included sex, race/ethnicity, age, body mass index, total energy intake, smoking, exposure to in-home smoking, pre-diabetes, diabetes, family income, education level, and total fruits and vegetables intake. They were selected for use in this study based upon existing research [9, 37–41]. Family income and education level were categorical variables used to adjust for socioeconomic status (SES). NHANES used the Family Interview Income Questionnaire to obtain combined family income for 13 income ranges. For analysis purposes these were reduced to 0 - $19,999; $20,000 – $34,999; $35,000 – $54,999; $55,000 and over. Education level was obtained by asking, “What is the highest grade or school level you have received?” Categories were < 9^th^ Grade; 9^th^ to 11^th^ Grade; $; HS/ GED; Some College; and College Graduate.

Total energy intake was based on the average of two 24-h recalls. Total energy intake and total fruit and vegetable intake were the only variables obtained from the 24-h dietary recall. Body mass index (BMI, Kg/m^2^) was calculated by the NHANES, from measured height and weight. Weight status was classified based on adult standardized cut-offs as follows: underweight/normal ≤ 24.99; overweight ≥ 25 and ≤ 29.99; and obese ≥ 30. Pre-diabetes and type 2 diabetes were assessed in the NHANES by measuring blood glycosylated hemoglobin, also known as hemoglobin A1c. Pre-diabetes was defined as A1c ≥ 5.7 % and ≤ 6.4 %. Diabetes was defined as A1c > 6.4 %.

Smoking and history of smoking was self-reported. On the NHANES questionnaire, this was asked as a series of questions including, “Do you now smoke cigarettes? During the past 5 days, did you use cigarettes?” A positive reply to either question was considered positive for smoking or history of smoking. Exposure to in-home smoking was self-reported. This was obtained by asking, “Does anyone smoke in the home.”

### Statistical analysis

Analysis was performed utilizing statistical software from STATA Corporation, revision 18. Appropriate procedures were used to account for the complex sample design. As previously described, weight variables were used to account for non-response and oversampling of various age groups and ethnic groups. Rao Scott Ҳ^2^ analysis was used to test for significance of differences in chronic bronchitis prevalence by intake frequency. A *p*-value of ≤ 0.05 was considered significant, with values <0.10 considered to approach significance.

Logistic regression was used to assess the adjusted odds between non-diet soft drinks intake and chronic bronchitis, independent of confounding variables. Two multivariate logistic regression models were used to analyze adjusted odds ratios (OR). The first model adjusted for age, sex, race/ethnicity, BMI, smoking, exposure to in-home smoking, pre-diabetes, and diabetes. The second model also adjusted for SES, total energy intake, and total fruit and vegetable intake – a potential confounder often used as an indicator of healthy lifestyle. In logistic regression analysis, confidence intervals that did not include 1 and *p* values ≤ 0.05 were considered statistically significant.

## Results

Overall, 6.1 % of 2,801 adults aged 20–55 y reported chronic bronchitis [or history of CB] Table 1. There was a statistically significant correlation between non-diet soft drink intake and prevalence and odds of CB in adults aged 20–55 y (*p* < 0.05). Unadjusted Rao Scott Ҳ^2^ comparisons with chronic bronchitis prevalence showed that non-diet soft drink intake was significantly associated with adult chronic bronchitis. Chronic bronchitis prevalence among ≥ 5 times a week non-diet soft drink (8.0 %) consumers was almost twice that of ≤ 1–3 a month (4.9 %) consumers, *P* = 0.041 Table 2.

**Table 2:** Unadjusted associations between Non-diet Soft Drink Intakes and Chronic Bronchitis in Adults aged 20–55 y in the NHANES 2003–2006

			95 %		Chronic	
	n	Proportions	Confidence		Bronchitis	
			Limits		% yes	*p*-value
	2801					
^a^Non-diet soft drinks						
≤1-3 times p/month		26.7 %	24.5-	29.0 %	04.9 %	0.041
1–4 times p/week		31.9 %	29.2-	34.8 %	04.6 %	
≥5 times or more p/week		41.3 %	37.9-	44.9 %	08.0 %	

^a^In 2003 – 2006 [the NHANES study period] high fructose corn syrup, a high excess free fructose sweetener, was the main sweetener in non-diet soda [23]

**Fig. 1:**
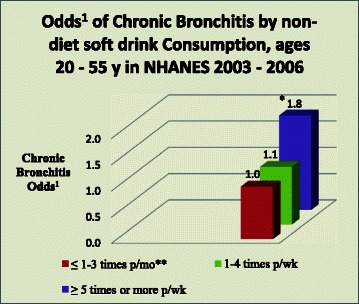
*OR 1.80, after adjustments; p-value = 0.047; 95 % CI 1.01-3.20; **reference group; ^1^Odds of Chronic Bronchitis by non-diet soft drink intake, adjusted for age, sex, BMI, race/ ethnicity, pre-diabetes, diabetes, smoking, exposure to in-home smoking, SES (socio-economic status), total energy intake and total fruit and vegetable intake; *N* = 1920. In 2003 – 2006 [the NHANES study period] HFCS was the main sweetener in U.S. non-diet soft drinks [25]

In multivariable logistic regression models, the correlation between non-diet soft drinks and chronic bronchitis was statistically significant. Adults aged 20–55 y reporting consumption of non-diet soft drinks ≥ 5 times a week had nearly two times the odds of chronic bronchitis as ≤ 1–3 a month non-diet soft drink consumers, after adjusting for age, sex, race/ ethnicity, BMI, total energy intake, smoking, exposure to in-home smoking, pre-diabetes and diabetes (OR = 1.89; p = 0.039; 95 % CI 1.03 – 3.45). Table 3. Adjustments for SES, total energy and total fruit and vegetable intake, a measure used as a barometer of healthy lifestyle, lowered the OR, but did not materially change the significance of the results (OR = 1.80; p = 0.047; 95 % CI 1.01 – 3.20). Fig. 1.

**Table 3:** Associations between Non-diet Soft Drink Intake and Chronic Bronchitis in Adults Aged 20–55 y, NHANES 2003–2006

	Multivariable	Logistic	Regression
	Model 1		
	ratio	95 % CI	*p*-value
OR – adjusted for sex, race/ ethnicity, age, BMI, smoking, exposure to in-home smoking, prediabetes, diabetes			
^a^Non-diet soft drinks			
≤1– 3 times/month	Reference		
1–4 times/week	1.06	0.51 – 2.21	0.875
≥5 times/week	1.89	1.03 – 3.45	0.039
	Subpop	n = 2801,	F (14, 17) = 6.75;
	Prob	> F = 0.0002	

^a^In 2003 – 2006 [the NHANES study period], high fructose corn syrup was the main sweetener in US non-diet soft drinks [23]

## Discussion

This study’s results support the hypothesis tested – increasing high fructose corn syrup sweetened soft drink consumption is significantly correlated with adult chronic bronchitis. Adults consuming non-diet soft drinks ≥ 5 times a week were nearly twice as likely to have chronic bronchitis as non/ low non-diet soft drink consumers, independent of all covariates, including smoking and history of smoking, *P* = 0.047. To our knowledge, this is the first US study to assess an association between soft drinks and chronic bronchitis. The results are consistent with another study, which showed an association between excess free fructose beverages consumption and pediatric asthma - a chronic childhood lung disease that significantly increases the risk of developing chronic bronchitis in adulthood [5]. Specifically, high consumption of excess free fructose beverages, including apple juice, fruit drinks and non-diet soda, was significantly correlated with asthma in children ages 2–9 y [10].

Results are similarly consistent with another recent study of mothers and their children. Children whose mothers drank high fructose corn syrup sweetened soft drinks during their first and second trimesters were 22 % more likely to have asthma as children whose mothers who did not drink non-diet soft drinks. Analyses with their children at ages 7–8 years showed that children, who drank juice *other than orange juice*, were 34 % more likely to have asthma than children who didn’t drink juice. It was suggested that early life exposure to fructose may influence asthma development in children [42].

However, their findings are more consistent with the conclusion that it is the excess free fructose that is associated with childhood asthma, because the association with asthma included consumption of high fructose corn syrup sweetened soft drinks by mothers during pregnancy, and included consumption of 100 % juice by their children, *exclusive of orange juice*. The conclusion that the asthma association is with fructose is less clear, because although the total fructose amounts in apple juice and orange juice are comparable, their excess free fructose contents are significantly different; per 100 g, apple juice contains 6.4 g and orange juice contains 4.5 g of fructose. [23]. In contrast, the excess free fructose content in an 8 oz cup of apple juice is 9.3 g (NDB No. 09400) and in orange juice it is 0.4 g (NDB No. 09207) [23].

Similarly, high fructose corn syrup sweetened soft drinks, as apple juice, contain significantly more excess free fructose than orange juice [16, 23, 43]. Average US per capita consumption of HFCS has been estimated to be just under 0.5 kg (1 lb.) per week, or approximately 65 g per day [44]. In HFCS that is made of 60 % fructose and 40 % glucose monomers [16], 65 g contains 13 g of excess free fructose. As one 20 oz bottle of cola contains 65 g of HFCS [45], a person who drinks 20 oz of cola sweetened with the 60/40 variant of HFCS, consumes 13 g of EFF in a single beverage serving. Notably, in fructose malabsorption research, 10 % of healthy adults challenged with 12 g of excess free fructose tested positive for fructose malabsorption [19–21].

In another recent cross-sectional asthma study, researchers identified a statistically significant association between increased non-diet soft drink consumption and asthma among high-schoolers [9]. It was suggested that preservatives might explain the correlation. However, given the results of existing epidemiology, their findings are more consistent with the possibility that the association with asthma is more likely to be attributed to the effects of excess free fructose.

The repeatability of EFF’s association with lung pathophysiology across age groups provides support for the hypothesis that increased excess free fructose consumption is associated with lung disease. In existing fructose malabsorption research, excess free fructose or just fructose is associated with fructose malabsorption, but not sucrose or equal amounts of fructose and glucose monomers [17–22]. This observation is consistent with the possibility that fructose malabsorption may underlie the association between excess free fructose consumption and lung disease.

Notably, the correlation between excess free fructose and chronic bronchitis was independent of co-morbidity factors often associated with COPD, including obesity, smoking, exposure to in-home smoking, pre-diabetes and diabetes. Existing research has provided evidence of elevated levels of AGEs/RAGE in COPD patients who underwent lobectomy [15]. Researchers suggested that the nexus between COPD and diabetes may be due to the elevated concentrations of AGEs/RAGE associated with diabetic hyperglycemia [15].

However, results of this study showed that the association between increased excess free fructose intake and chronic bronchitis was independent of pre-diabetes and diabetes, suggesting that the link between increased excess free fructose consumption and chronic bronchitis is independent of the glycemic load thought to contribute to AGEs formation in the systemic circulation of individuals with diabetes. Our results support the possibility that the in situ formation of enFruAGEs, resulting from the interaction between unabsorbed excess free fructose and dietary proteins, may be a source of elevated levels of advanced glycation end-products in COPD patients, independent of diabetes.

Equally noteworthy is that the association between increased excess free fructose consumption and higher odds of chronic bronchitis was independent of smoking. This suggests that high excess free fructose consumption may, in part, explain the idiopathic prevalence of chronic bronchitis among non-smokers. Results are consistent with and provide support for recent research that suggests that smoking associated emphysema and non-smoking associated chronic bronchitis - entities that are traditionally considered to be overlapping within COPD - may in fact be distinct disorders [7].

It is interesting that a 2008 population-based study found that COPD patients were more likely to have symptoms of inflammatory bowel disease than history of smoking [26]. The recent evidence that intestinal distress is increased in COPD patients [24–26] is consistent with, and provides further support for, the “intestinal enFruAGEs fructositis” hypothesis. Research indicating that AGEs are elevated in lung tissues of COPD patients [14] and that overexpression of RAGE and AGEs was observed in bronchiolar epithelia, type II alveolar pneumocytes, alveolar macrophages and endothelia in pathological conditions associated with inflammation and lung damage [11] should be considered in the context of evidence that intestinal compromise is a newly identified component of COPD [27]. The suggestion that COPD is a multisystem disorder that involves aberrant intestinal symptomology [27] is consistent with this study’s results.

Reports by the American Lung Association indicate that children who suffered from severe, persistent asthma are nearly 32 times more likely to develop COPD in adulthood [6]. The statistically significant correlations between increased excess free fructose consumption and prevalent adult chronic bronchitis - and increased excess free fructose consumption and prevalent pediatric asthma [10] - support the possibility that excess free fructose and enFruAGE are risk factors in lung disease across age groups.

This study is subject to limitations. First, the associations are cross-sectional; therefore, the exposures and outcome are simultaneously assessed. For this reason, cross-sectional studies do not provide evidence of a temporal relationship between exposures and outcome. Follow-up longitudinal studies are needed. Second, chronic bronchitis status in NHANES is based on self-report, so there is potential for reporting bias. Third, other possible confounders for COPD and chronic bronchitis, including environmental factors, such as outdoor air quality, could not be accounted for because these data were not available. Fourth, high excess free fructose beverages are only one food category that could contribute to daily excess free fructose load. Numerous other food categories contain HFCS as an added sweetener, and therefore, also contribute to daily excess free fructose load. However, among US adults, soft drinks are the most significant source of high fructose corn syrup in the American diet [46].

## Conclusion

Increasing excess free fructose consumption is significantly correlated with adult chronic bronchitis. This study’s results provide epidemiological support for the association between consumption of high fructose corn syrup sweetened soft drinks, in the possible pathogenesis of chronic bronchitis – a subcomponent of COPD. The previously proposed “intestinal enFruAGEs fructositis” hypothesis - that underlying fructose malabsorption and associated fructose reactivity in the GI may contribute to intestinal in situ formation of enFruAGEs that travel to the lungs provides a possible biological mechanism for this association. [8] Longitudinal, clinical, and biochemical research is needed to confirm and clarify the mechanisms involved.

## Abbreviations

AGEs: Advanced glycation end-products
ALA: American Lung Association
BMI: Body mass index
CB: Chronic bronchitis
COPD: Chronic obstructive pulmonary disease
EFF: Excess free fructose
enFruAGE: Extracellular, newly identified, fructose associated advanced glycation end-product
FFQ: Food frequency questionnaire
FM: Fructose malabsorption
GI: Gastro intestinal
HFCS: High fructose corn syrup
NDB: US National nutrient database for standard reference
NHANES: National Health and Nutrition Examination Survey
RAGE: Receptor of advanced glycation end-products
SES: Socioeconomic status
